# 3‴-*O*-Foliamenthoyl-Rutin, a New Flavonoid Glycoside from the Roots of *Nymphoides peltata*

**DOI:** 10.3390/plants12244083

**Published:** 2023-12-06

**Authors:** Tae-Young Kim, Bum Soo Lee, Beom-Geun Jo, Seong Pil Heo, Min-Ji Keem, Taek-Hwan Kwon, Su-Nam Kim, Ki Hyun Kim, Min Hye Yang

**Affiliations:** 1 Department of Pharmacy, College of Pharmacy and Research Institute for Drug Development, Pusan National University, Busan 46241, Republic of Korea; taeyour@pusan.ac.kr (T.-Y.K.); bg_jo@pusan.ac.kr (B.-G.J.); mj_keem@pusan.ac.kr (M.-J.K.); thkwon@pusan.ac.kr (T.-H.K.); 2 School of Pharmacy, Sungkyunkwan University, Suwon 16419, Republic of Korea; kosboybs@skku.edu; 3 Natural Products Research Institute, Korea Institute of Science and Technology, Gangneung 25451, Republic of Korea; 123045@kist.re.kr; 4 Division of Bio-Medical Science and Technology, KIST School, University of Science and Technology, Seoul 02792, Republic of Korea

**Keywords:** *Salix chaenomeloides*, phenolic glycoside, chaenomelin, *Helicobacter pylori*

## Abstract

*Nymphoides peltata* (Menyanthaceae) has been used as a medicinal herb in traditional medicines to treat conditions such as strangury, polyuria, swelling, and as a diuretic and antipyretic. In our ongoing research to discover novel structural and/or biological natural products in natural resources, five flavonoids, quercetin (**1**), quercitrin (**2**), isoquercetin (**3**), quercetin-3-*O*-vicianoside (**4**), and rutin (**5**), as well as a new flavonoid glycoside, 3‴-*O*-foliamenthoyl-rutin (**6**), were isolated from the MeOH extract of *N. peltata* roots. The chemical structure of the new compound (**6**) was determined by analyzing 1D and 2D NMR spectra and high-resolution (HR) electrospray ionization mass spectroscopy (ESIMS), along with a chemical reaction. The wound-healing activities of the isolated compounds (**1**–**6**) were evaluated using a HaCaT cell scratch test. Among the isolates, isoquercetin (**3**), quercetin-3-*O*-vicianoside (**4**), and 3‴-*O*-foliamenthoyl-rutin (**6**) promoted HaCaT cell migration over scratch wounds, with compound **4** being the most effective. Our findings provide experimental data supporting the potential of quercetin-3-*O*-vicianoside (**4**) as a wound-healing agent.

## 1. Introduction

Menyanthaceae is a family of perennial aquatic plants that consists of approximately 60 species in 6 genera [1]. The genus *Nymphoides* is the largest within Menyanthaceae, comprising around 55 species [2]. These plants are native to temperate and subtropical regions, with endemism in Korea, China, and Japan [3,4]. Phytochemical studies on the *Nymphoides* genus have revealed the presence of polyphenols, flavonoids, triterpenes, and ferulic acid as major compounds. Furthermore, these plants exhibit various pharmacological activities, including anticonvulsant, antioxidant, and antidiabetic effects [5,6,7,8]. *Nymphoides peltata*, also known as the yellow floating heart, is a medicinal herb with historical use in traditional Chinese medicines like Ben Cao Gang Mu, as well as in traditional Indian medicines like Ayurveda. It is employed to treat conditions such as heat strangury and polyuria, as a diuretic or antipyretic, and for addressing swelling [5]. In a recent pharmacological study, an MTT assay revealed that a 95% ethanol extract of *N. peltata* exhibited significant antitumor activity against prostate cancer (PC3) and osteosarcoma cells (U2OS) [9]. Despite the numerous studies reporting the pharmacological activity of *N. peltata* extract, limited research has been conducted on its phytochemical composition at the species level.

The skin, being the largest organ of humans, serves as an essential physical shield against harmful pathogens and radiation, playing a pivotal role in maintaining life on an ongoing basis. However, this robust defense can be compromised by various forms of trauma, such as battles, bites, thermal burns, car accidents, and more, leading to breaks in the continuity of the skin [10,11]. Healing these skin injuries is a complex and dynamic process, remaining a significant clinical challenge. Over the years, numerous techniques have been proposed to facilitate the healing of skin injuries, including the application of ointments, the use of specialized wound dressings, and even graft transplantation to ensure proper tissue repair [12,13]. However, synthetic drugs, despite their efficacy, come with a high risk of side effects, including allergic reactions and the development of drug resistance. In light of these concerns, natural compounds have emerged as a powerful alternative in the field of wound-healing medicine [14]. Alternative herbal medicine, in particular, has gained prominence in the context of skin injury healing. This approach emphasizes moist wound healing, eliminating the need for prolonged dressing, and introducing a more efficient and patient-friendly healing process [15]. Additionally, certain herbs used in this alternative medicine have been recognized for their remarkable antimicrobial properties, which can effectively combat bacteria and prevent infection in wounds [16]. Thus, the use of natural compounds and herbal remedies not only promises effective wound management but also offers a safer and more sustainable approach.

Wound healing is a sophisticated process that commences following an injury and unfolds through the well-orchestrated interplay of various pathways, growth factors, and a diverse array of cell and tissue types. The pattern of wound healing is characterized by the gradual contraction of the wound area, facilitated by the inward movement of the wound border toward the wound core, ultimately leading to wound closure. The pursuit of medications that can accelerate wound healing and potentially contribute to all phases of the healing process remains imperative for effective treatment. These medications should ideally be cost-effective and entail fewer side effects, underscoring the importance of exploring natural compounds in this context.

Polyphenolic compounds, with a particular emphasis on flavonoids, are ubiquitous constituents found throughout the plant kingdom. These compounds have earned recognition for their remarkable anti-inflammatory properties and their role in promoting wound healing [17]. Due to their potent anti-inflammatory effects, certain flavonoids have emerged as significant players in the realm of wound healing, demonstrating their efficacy in both in vitro and in vivo models. Among the notable flavonoids with documented roles in wound healing are apigenin, rutin, and catechin [17,18]. These compounds have showcased their potential to facilitate the wound-healing process. Additionally, sativanone-7-*O*-glucoside and ononin have been identified as contributors to wound healing, further expanding the roster of flavonoids known for their healing properties [19]. Prunetin and genistein are also recognized for their wound-healing capabilities, reflecting the diversity of flavonoids with therapeutic potential [20]. The list of wound-healing flavonoids extends to include luteolin, linarin, and 6-hydroxyluteolin, which have exhibited promising effects in facilitating the healing of various types of wounds [21]. Kaempferol and vicenin-2 are additional flavonoids that have demonstrated their potential as valuable agents in the wound-healing process [22]. The wealth of evidence surrounding these polyphenolic compounds, particularly flavonoids, underscores their multifaceted role as potent anti-inflammatory and wound-healing agents. Their presence in the plant kingdom offers a rich source of natural remedies with the potential to revolutionize wound care and bring about more effective and sustainable approaches to healing. As research in this field continues to advance, the healing properties of these compounds may pave the way for innovative and accessible wound-healing therapies.

In this study, we directed our focus toward investigating the presence of new bioactive metabolites in the methanol (MeOH) extract of *N. peltate* root. In our recent study of *N. peltate* root, it was demonstrated that a 95% EtOH extract of *N. peltata* root inhibited IL-4 expression in PMA/ionomycin-induced RBL-2H3 cells and exhibited anti-atopic effects in oxazolone- and 2,4-dinitrochlorobenzene (DNCB)-induced mouse models [23]. In the present study of *N. peltate* root, which is aimed at identifying new secondary metabolites, the MeOH extract of *N. peltate* root was investigated through extensive chromatographic purifications and LC/MS analysis in conjunction with the references of an in-house UV spectra library. As a result, we successfully identified and isolated six flavonoids (**1**–**6**), including a new flavonoid glycoside (**6**). The chemical structure of the new compound (**6**) was unequivocally elucidated using a combination of 1D and 2D NMR spectroscopic techniques, high-resolution (HR) electrospray ionization mass spectroscopy (ESIMS), and a chemical reaction. Further, we evaluated the isolated compounds (**1**–**6**) for their wound-healing activities using a HaCaT cell scratch test. Here, we report the isolation and structural characterizations of the compounds isolated, as well as their wound-healing activities.

## 2. Results and Discussion

### 2.1. Isolation and Structural Elucidation of Compounds ***1**–**6***

**Figure 1:**
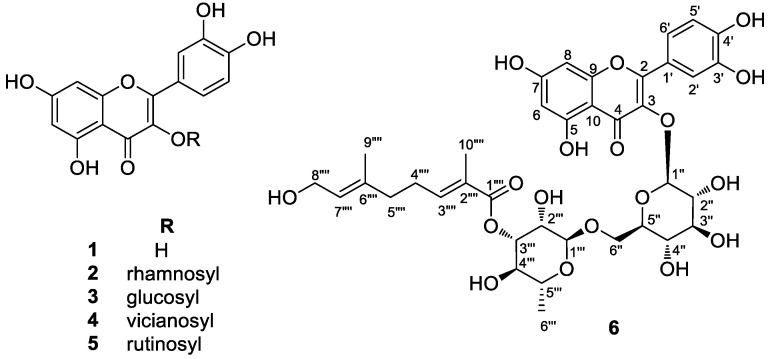
Chemical structures of compounds **1**–**6**.

The roots of *N. peltata* were extracted with methanol (MeOH), and the resulting MeOH extract was fractionated using solvent partitioning with *n*-hexane (Hx), ethyl acetate (EtOAc), and *n*-butanol (BuOH) to obtain three major fractions. Column chromatography procedures, including open-column chromatography, Sephadex LH-20, and reverse-phase HPLC purification, together with LC/MS analysis in conjunction with reference to an in-house UV spectra library, were applied to the EtOAc fractions. This process led to the isolation of six flavonoid derivatives (**1**–**6**) (Figure 1). The chemical structures of the isolated compounds were definitively determined to be quercetin (**1**) [24], quercitrin (**2**) [25], isoquercetin (**3**) [24], quercetin-3-*O*-vicianoside (**4**) [26], and rutin (**5**) [24] through comparison and validation of their NMR spectroscopic data with those previously reported and high-resolution electrospray ionization mass spectrometry (HR-ESI-MS) analyses (Table S1). Among the isolated compounds, compound **6** was identified as a new flavonoid glycoside derivative.

Compound **6** was isolated as a yellowish amorphous powder. The molecular formula was confirmed to be C_37_H_44_O_18_ based on the proton-adducted molecular ion peak [M + H]^+^ *m/z* 777.2561 (calculated for C_37_H_45_O_18_, 777.2606) from the positive-ion high-resolution electrospray ionization mass spectrometry (Figure S1). The IR spectrum exhibited absorption bands corresponding to hydroxyl (3400 cm^−1^), carbonyl (1655 cm^−1^), and aromatic (1515 cm^−1^) functionalities. The ^1^H NMR data (Table 1, Figure S2) of **6** showed the presence of proton signals for H-6 [*δ*_H_ 6.16 (1H, d, *J* = 2.0 Hz)] and H-8 [*δ*_H_ 6.36 (1H, d, *J* = 2.0 Hz)] of the A-ring and H-5′ [6.84 (1H, d, *J* = 8.0 Hz)], H-2′ [*δ*_H_ 7.52 (1H, d, *J* = 8.0, 2.0 Hz)], and H-6′ [7.53 (1H, dd, *J* = 8.0, 2.0 Hz)] of the B-ring in the flavonoid backbone. The characteristic proton signals suggested that the flavonoid skeleton is a quercetin, which was also supported by the ^13^C NMR (Table 1, Figure S3) data of **6**, showing 15 carbon signals corresponding to quercetin aglycone, including one carboxyl carbon (*δ*_C_ 177.8) [24]. In addition, two anomeric NMR signals for the sugar units were observed at *δ*_H_ 5.34 (1H, d, *J* = 7.0 Hz)/*δ*_C_ 101.8 and at *δ*_H_ 4.41 (1H, br s)/*δ*_C_ 101.3, and the sugar units were assigned to rutinose (6-*O*-*α*-L-rhamnosyl-D-glucose) composed of glucose (*δ*_C_ 101.8, 76.9, 76.3, 74.5, 71.8, 68.0) and rhamnose (*δ*_C_ 101.3, 74.7, 69.4, 68.9, 68.3, 18.1) using the ^13^C NMR (Table 1) data of the sugar units [24] that were obtained with the assistance of the HSQC experiment (Figure S5). Based on this information, compound **6** was determined to possess a rutin moiety [24].

**Figure 2:**
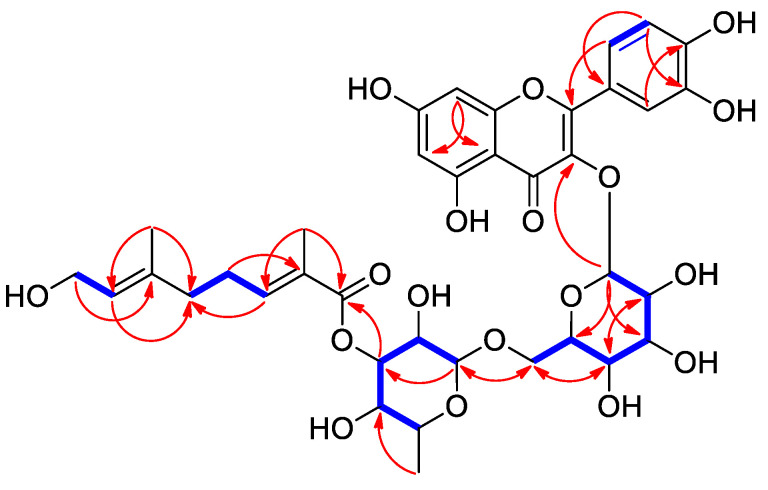
Key ^1^H-^1^H COSY (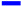) and HMBC (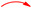) correlations for compound **6**.

Additionally, interesting signals assignable to a monoterpene unit [*δ*_H_ 6.70 (1H, t, *J* = 6.5 Hz, H-3⁗), 5.31 (1H, t, *J* = 6.5 Hz, H-7⁗), 3.94 (2H, d, *J* = 6.5 Hz, H-8⁗), 2.25 (2H, dt, *J* = 6.5, 7.0 Hz, H-4⁗), 2.07 (2H, t, *J* = 7.0 Hz, H-5⁗), 1.77 (3H, s, H-10⁗), and 1.61 (3H, s, H-9⁗), and *δ*_C_ 167.3 (C-1⁗), 142.0 (C-3⁗), 135.6 (C-6⁗), 128.1 (C-2⁗), 126.1 (C-7⁗), 58.0 (C-8⁗), 38.0 (C-5⁗), 27.1 (C-4⁗), 16.5 (C-9⁗), and 12.8 (C-10⁗)] were observed. Collectively, the NMR spectra (Table 1) of **6** were almost identical to those of rutin (**5**) [24], with the only difference being the presence of a monoterpene group. The monoterpene group was determined to be a foliamenthoyl moiety [27] based on two methyl groups at *δ*_H_ 1.61 (3H, s, H-9⁗) and *δ*_H_ 1.77 (3H, s, H-10⁗), two olefinic protons at *δ*_H_ 6.70 (1H, t, *J* = 6.5, H-3⁗) and *δ*_H_ 5.31 (1H, t, *J* = 6.5, H-7⁗), and one oxygenated methylene proton at *δ*_H_ 3.94 (2H, d, *J* = 6.5 Hz, H-8⁗), as well as one carboxyl carbon signal (*δ*_C_ 167.3). The foliamenthoyl moiety was also confirmed with the analysis of the ^1^H-^1^H COSY correlations of H-3⁗/H-4⁗/H-5⁗ and H-7⁗/H-8⁗ and the HMBC correlations of H-9⁗/C-5⁗, H-9⁗/C-7⁗, H-8⁗/C-6⁗, H-10⁗/C-1⁗, H-10⁗/C-3⁗, and H-4⁗/C-2⁗ (Figure 2). The location of the foliamenthoyl moiety was established at C-3 of rhamnose through a relatively deshielded proton signal at *δ*_H_ 4.63 for H-3 of rhamnose and the HMBC correlation of H-3 of rhamnose (*δ*_H_ 4.63)/C-1⁗ (*δ*_C_ 167.3) (Figure 2). Further detailed analysis of the HMBC (Figure S6) and ^1^H-^1^H COSY spectra (Figure S4 and Figure 2) revealed the complete planar structure of **6**, as shown in Figure 1. Finally, the coupling constants of the anomeric protons at *δ*_H_ 5.34 (1H, d, *J* = 7.0 Hz) and *δ*_H_ 4.41 (1H, br s) were confirmed as *β*-glucopyranose and *α*-rhamnopyranose, and acid hydrolysis of compound **6** gave D-glucopyranose and L-rhamnopyranose, which was confirmed using LC/MS analysis [28]. Therefore, based on the obtained data, compound **6** was elucidated as the new flavonoid glycoside shown in Figure 1 and named 3‴-*O*-foliamenthoyl-rutin.

### 2.2. Wound-Healing Activities of Compounds ***1**–**6***

**Figure 3:**
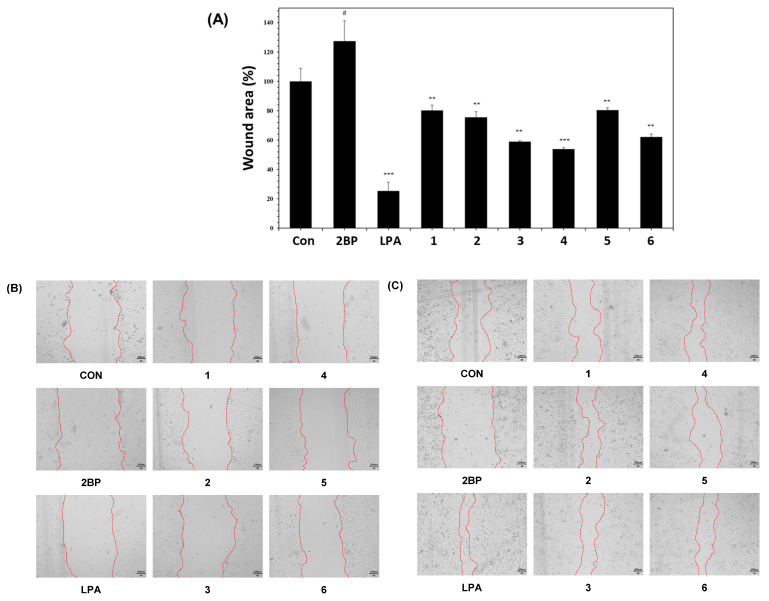
Effects of compounds **1**–**6** on HaCaT cell wound-healing assay results. Scratched HaCaT monolayers were treated with 10 μM of 2BP, LPA, or compounds **1**–**6**, and cell migration was observed under a microscope after 48 h of culture (**A**). Wound closure was monitored (**B**) and wound spacing was measured (**C**). Each bar is presented as mean ± SD (*n* = 3). Wound area photographs and wound area over time (^#^ *p* < 0.05 vs. CON; *** *p* < 0.001, ** *p* < 0.005 vs. 2BP). CON: untreated group, 2BP: 2-bromo-palmitate-treated group, LPA: lysophosphatidic acid-treated group.

Keratinocytes are essential constituents of the epidermis and play a pivotal role in the regeneration of the epidermis during wound healing, encompassing processes like hemostasis, inflammation, proliferation, and remodeling [29,30]. The wound-healing activities of the compounds **1**–**6** isolated from *N. peltata* were evaluated using a HaCaT cell scratch test, an in vitro method that proves valuable for assessing cell migration from wound edges and over scratches [31]. The negative control, 2-bromo-palmitate (2BP), resulted in reduced cell migration, increasing the wound area to 124.04%. As a positive control, lysophosphatidic acid (LPA), known for its role in epidermal regeneration by modulating cellular responses [32], decreased the wound area to 23.97%. Furthermore, our results revealed that HaCaT cells exhibited enhanced wound healing in the presence of isoquercetin (**3**) (reducing the wound area to 66.48%), quercetin-3-*O*-vicianoside (**4**) (reducing the wound area to 61.08%), and 3‴-*O*-foliamenthoyl-rutin (**6**) (reducing the wound area to 71.42%) (Figure 3). Among these active compounds, quercetin-3-*O*-vicianoside (**4**) proved to be the most effective in promoting wound repair.

Quercetin (**1**), a bioactive flavonoid often found in the form of quercetin glycoside, is notable for its safety within reasonable dosage ranges [33]. It boasts a diverse range of healing properties, including anti-inflammatory, anti-infectious, angiogenic, immune-regulating, and antioxidant attributes [33]. When applied topically, quercetin demonstrates the ability to elevate interleukin-10 levels (an anti-inflammatory cytokine) while reducing tumor necrosis factor-*α* (TNF-*α*), an inflammatory factor [33]. Furthermore, quercetin exhibits promise in wound healing by increasing levels of vascular endothelial growth factor (VEGF), transforming growth factor-β (TGF-β), and antioxidants, while simultaneously diminishing the expression of inflammatory elements [33]. Despite these positive effects, a therapeutic effect of quercetin in healing wounds was not found in the current study. Quercitrin (**2**) has been reported to possess in vitro wound-healing capabilities. Previous studies have highlighted the involvement of the Wnt/*β*-catenin signaling pathway for quercitrin in enhancing wound healing by regulating cell proliferation [34]. Quercitrin was found to enhance motility in HaCaT keratinocytes by increasing *β*-catenin levels and activating the Wnt/*β*-catenin signaling pathway [34]. This discovery indicates its potential in promoting wound healing by facilitating cellular processes. Furthermore, quercitrin demonstrated non-cytotoxic effects under therapeutic concentrations (10 μM), suggesting its suitability as a potential therapeutic agent for wound healing. However, the observed therapeutic concentration of quercitrin (10 μM) did not demonstrate the expected effects in wound healing in the current study. In the case of isoquercetin (**3**), wound-healing activity was confirmed in isoquercetin that was isolated sequentially through sequential fractionation, beginning with the *Sambucus ebulus* extract from a previous study [35]. However, it was noted that the independent activity of isoquercetin, which was identified as the primary single compound in the active extract with confirmed wound-healing activity, was significantly lower than that of the subfractions [35]. Those results were partially consistent with our findings, wherein compound **3** exhibited weak activity. Contrastingly, quercetin-3-*O*-vicianoside (**4**), which is characterized by different sugar units, demonstrated activity in wound healing in our bioassay system, marking the first report of such activity in the current study. In the case of rutin (**5**), previous experiments were conducted to evaluate the relationship between wound healing in streptozotocin-induced hyperglycemic rats and the antioxidant and anti-inflammatory effects of rutin [36]. Eighteen male Wistar rats were randomly divided into three groups: normal, hyperglycemic, and rutin-containing hyperglycemic groups. After inducing hyperglycemia for 2 days, a wound was induced on the back of each rat. It was confirmed that intraperitoneal injection of rutin significantly improved diabetes-induced weight loss and alleviated metabolic dysfunction in hyperglycemic rats [36]. Finally, the new compound identified in this study, 3‴-O-foliamenthoyl-rutin (**6**), demonstrated the ability to promote HaCaT cell migration over scratch wounds, indicating its potential as a wound-healing agent.

Normal wound healing is a dynamic and complex process that involves a series of coordinated events, including bleeding, coagulation, initiation of an acute inflammatory response to the initial injury, and subsequent regeneration, migration, and proliferation of connective tissue, along with collagen deposition. Among these events, cell migration and proliferation play crucial roles in wound healing. The wound site progresses through various stages, relying on cellular processes such as collagen synthesis, angiogenesis, granulation tissue formation, and epithelialization to undergo recovery [37]. 

In this study, we investigated the effects of compounds **1**–**6** on cell migration and proliferation in HaCaT cells, which are keratinocytes. Our findings revealed that compounds **3**, **4**, and **6** positively influence wound healing. Consequently, additional experiments are deemed necessary to validate whether changes in concentration contribute to wound healing. If wound healing is concentration-dependent, further research on the various mechanisms mentioned above would be warranted.

## 3. Materials and Methods

### 3.1. General Experimental Procedures

Nuclear magnetic resonance (NMR) spectra analysis (^1^H, ^13^C, ^1^H-^1^H COSY, HMBC, and HMQC) spectra were observed on a JEOL JNM-ECZ400S 400 MHz NMR spectrometer (JEOL, Ltd., Tokyo, Japan) and Bruker AVANCE NEO 500 MHz NMR spectrometer (Bruker Corp., Billerica, MA, USA). HR-ESI-MS was measured using Agilent 6530 Accurate-Mass Q/TOF-LC/MS system (Agilent Technologies, Santa Clara, CA, USA). The high-performance liquid chromatography–photodiode array (HPLC-PDA) analysis was performed on the Waters HPLC system with an e2695 separation module and a 2998 PDA detector (Waters Corporation, Milford, MA, USA) and Empower^®^3 Chromatography Software version 3 (Build 3471, Waters Corp., MA, USA) and a reversed-phase column (Aegispak C18-L, 5 μm, 4.6 × 250 mm, Young Jin Biochrom, Sungnam, Republic of Korea). Semipreparative HPLC was performed on a Gilson HPLC system with two pumps (305 master pump, 307 slave pump) and a mixer (811C dynamic mixer) and a Shimadzu HPLC system with a pump (LC-20AT), UV/vis detector (SPD-20A), and system controller (CBM-20A) using the reversed-phase column (Watchers 120 ODS-BP, S-10 μm, 150 × 10 mm, Isu Industry Corp., Seoul, Republic of Korea). The resin for open-column chromatography used Silica gel 60 with pore size of 6 nm and particle size of 63–200 µm (Product No. 1.07734, Merck, Darmstadt, Germany) and Sephadex™ LH-20 with bead size of 25–100 µm (GE Healthcare Bio-Sciences AB, Uppsala, Sweden). The gel-coated plate used in the thin-layer chromatography (TLC) detection assay was silica gel 60 F254 Art (GF254, No. 1.05554, Merck, Darmstadt, Germany), and spots were observed using UV spectrum and anisaldehyde–sulfuric acid reagent.

### 3.2. Plant Material

The root parts of *Nymphoides peltate* (Gmel.) O. Kuntze were collected in the Hantaek Botanical Garden Foundation, Yongin-si, Gyeonggi-do, Republic of Korea, in June 2021. The plants were authenticated by Dr. Jung Hwa Kang. A voucher specimen (PNU-0040) was deposited in the Medicinal Herb Garden, Pusan National University.

### 3.3. Extraction and Isolation

The dried and powdered root parts of *N. peltata* (2.8 kg) were ultrasonically extracted twice with MeOH (28 L, 90 min each) at room temperature. The solvent was concentrated under reduced pressure at 45 °C and immediately freeze dried to obtain the crude *N. peltata* extract (427 g, yield: 15.2%). The crude extract obtained was suspended in H_2_O (2 L) and then partitioned with hexane (Hx, 8 L), ethyl acetate (EtOAc, 8 L), and *n*-butanol (BuOH, 8 L) to obtain three fractions, including soluble fractions of Hx (80.3 g), EtOAc (24 g), and BuOH (108 g). The soluble fraction of EtOAc was subjected to normal-phase silica column chromatography using a gradient mobile phase (EtOAc:MeOH from 10:1 to 100% MeOH) to obtain 7 subfractions (E1~E7). E1 (463.2 mg) was separated into 9 subfractions (E1-1~E1-9) with normal-phase silica column chromatography using a gradient mobile phase (EtOAc:MeOH from 20:1 to 100% MeOH). E1-6 (30.5 mg) was subjected to Sephadex LH-20 using 100% MeOH to obtain compound **1** (3 mg). E2 (1.025 g) was separated into 9 subfractions (E2-1~E2-9) with normal-phase silica column chromatography using a gradient mobile phase [chloroform (CHCl_3_):MeOH from 20:1 to 100% MeOH]. E2-9 (206 mg) was separated into 3 subfractions (E2-9-1~E2-9-3) with Sephadex LH-20 using 100% MeOH. E2-9-2 (75 mg) was separated into 3 subfractions (E2-9-2-1~E2-9-2-3) with Sephadex LH-20 using 100% MeOH. E2-9-2-3 (18.7 mg) was subjected to a Shimadzu preparative-HPLC system (UV wavelengths at 250 and 330 nm; flow rate of 2 mL/min) using an isocratic mobile phase (0.1% formic acid + MeCN:0.1% formic acid + H_2_O = 25:75) to obtain compound **2** (8.8 mg, *t_R_* = 13.2 min). E3 (1.1036 g) was separated into 6 subfractions (E3-1~E3-6) with Sephadex LH-20 using a 100% MeOH. E3-2 (357 mg) was separated into 5 subfractions (E3-2-1~E3-2-5) with normal-phase silica column chromatography using a gradient mobile phase (EtOAc:MeOH from 20:1 to 100% MeOH). E3-2-3 (137.9 mg) was separated into 3 subfractions (E3-2-3-1~E3-2-3-3) with Sephadex LH-20 using a 100% MeOH. E3-2-3-2 (67.8 mg) was subjected to a Shimadzu preparative-HPLC system (UV wavelengths at 250 and 330 nm; flow rate of 2 mL/min) using an isocratic mobile phase (0.1% formic acid + MeCN:0.1% formic acid + H_2_O = 25:75) to obtain compound **6** (31 mg, *t_R_* = 35 min). E7 (1.893 g) was subjected to a Shimadzu preparative-HPLC system (UV wavelengths at 250 and 330 nm; flow rate of 2 mL/min) using an isocratic mobile phase (0.1% formic acid + MeCN:0.1% formic acid + H_2_O = 20:80) to obtain compounds **4** (20.9 mg, *t_R_* = 18 min), **5** (22.4 mg, *t_R_* = 22 min), and **3** (21.7 mg, *t_R_* = 27.5 min).

#### 3‴-*O*-Foliamenthoyl-Rutin (**6**)

Yellowish amorphous powder; ${\left[\alpha \right]}_{D}^{25}$+15.8(*c* 0.02, MeOH); UV (MeOH) *λ*_max_ 254, 354 nm (Figure S7); IR (KBr) *ν*_max_ 3400, 2925, 2715, 1655, 1605, 1515, 1380, 1015 cm^−1^; ^1^H (500 MHz, DMSO-*d*_6_) and ^13^C (100 MHz, DMSO-*d*_6_) NMR data, see Table 1; HR-ESI-MS (positive ion mode) *m/z* 777.2561 [M + H]^+^ (calcd. for C_37_H_45_O_18_, 777.2606).

### 3.4. Acid Hydrolysis and Absolute Configuration Determination of Sugar Moieties

The absolute configuration of the sugar moieties was determined using an HPLC-UV-based method [28,38,39,40]. For compound **6**, 1.5 mg was hydrolyzed in the presence of 1 N HCl at 80 °C for 2 h and EtOAc was used for the extraction. The aqueous layer was neutralized with repeated evaporation under a vacuum evaporator and dissolved in anhydrous pyridine (0.5 mL) with the addition of L-cysteine methyl ester hydrochloride (1.0 mg). After the reaction mixture was heated at 60 °C for 1 h, *o*-tolylisothiocyanate (50 μL) was added and the mixture was kept at 60 °C for 1 h. The reaction product was evaporated under a vacuum evaporator and dissolved in MeOH. Next, the dissolved reaction product was directly analyzed with LC/MS [MeOH/H_2_O, 1:9 → 7:3 gradient system (0–20 min), 100% MeOH (21–31 min), 0% MeOH (32–42 min); flow rate of 0.3 mL/min] using an analytical Kinetex C_18_ 100 Å column (100 mm × 2.1 mm i.d., 5 μm). The sugar moieties from **6** were identified as *β*-glucopyranose and *α*-rhamnopyranose based on comparison with the standard using LC/MS analysis.

### 3.5. Cell Culture

Human keratinocytes (HaCaT) were purchased from CLS Cell Lines Service GmbH (Eppelheim, Baden-Württemberg, Germany) and cultured as single layers at 37 °C in a 5% CO_2_ incubator (Forma Direct Heat CO_2_ Incubator, Thermo Fisher Scientific, Madison, WI, USA) in DMEM (HyClone; Cytiva, Washington, DC, USA) supplemented with 10% fetal bovine serum (FBS; Gibco, Grand Island, NY, USA), 100 U/mL penicillin, and 100 μg/mL streptomycin (Gibco, Grand Island, NY, USA) (growth medium). 

### 3.6. Wound-Healing Assay and MTT Assay

The wound-healing assay was performed as previously described [31] with some modifications. HaCaT cells were seeded in 24-well plates at a density of 2 × 10^5^ cells per well and allowed to attach for one day. Scratches were then created in each well using a 200 μL pipette tip. After washing with PBS, cells were treated with 2-bromo-palmitate (2BP) at 10 μM (3.3532 μg/mL) or lysophosphatidic acid (LPA) at 10 μM (4.3652 μg/mL) as negative and positive controls, respectively, and compounds **1**–**6** at 10 μM (**1**: 3.0224 μg/mL, **2**: 4.4838 μg/mL, **3**: 4.641 μg/mL, **4**: 5.965 μg/mL, **5**: 6.1052 μg/mL, and **6**: 7.76 μg/mL). In addition, samples were cultured in a growth medium containing 1% FBS and 1% penicillin-streptomycin for 48 h to minimize the effects of growth factors. Wound images were captured immediately after wounding and after 24 or 48 h of culture using a microscope camera, HK5.1 UCMOS (KOPTIC, Republic of Korea). Captured images were quantified using Image J 1.52a software (National Institutes of Health, Bethesda, MD, USA). The wound area was recorded through phase-contrast imaging using an optical microscope. For quantification using Image J, the wound area was manually measured at both the 0 h and 48 h time points for each dataset. The results were expressed as the efficiency of wound closure, calculated as [(sample wound area/control wound area) × 100%].

### 3.7. Statistics Analysis

Data were expressed as the means ± standard deviation. GraphPad Prism, version 5.04 (GraphPad Software Inc., San Diego, CA, USA) was used for statistical processing in this experiment. The differences between the experimental groups were analyzed through the ANOVA test, and the significant level difference was set to *p* < 0.05.

## 4. Conclusions

A new flavonoid glycoside, 3‴-*O*-foliamenthoyl-rutin (**6**) and five known flavonoids, namely quercetin (**1**), quercitrin (**2**), isoquercetin (**3**), quercetin-3-*O*-vicianoside (**4**), and rutin (**5**), were isolated from the MeOH extract of *N. peltata* roots. The chemical structure of the newly isolated compound (**6**) was determined via 1D and 2D NMR spectra, HR-ESIMS analysis, and a chemical reaction. In our evaluation of the wound-healing activities of the isolated compounds **1–6** using a HaCaT cell scratch test, isoquercetin (**3**), quercetin-3-*O*-vicianoside (**4**), and 3‴-*O*-foliamenthoyl-rutin (**6**) promoted HaCaT cell migration over scratch wounds. Quercetin-3-*O*-vicianoside (**4**) proved to be the most effective in promoting wound repair. Our findings provide supportive data for the potential of quercetin-3-*O*-vicianoside (**4**) as a wound-healing agent. It is essential to acknowledge the limitations of our study. The biological evaluation, which was conducted with a minimum of three repetitions and a single concentration, may constrain the validity of our conclusions. Therefore, future studies are warranted to further investigate and substantiate the implications of our findings. 

## Figures and Tables

**Table 1:** ^1^H and ^13^C NMR data for compound **6** in DMSO-*d*_6_ (*δ* ppm) ^a^.

Position	6	
	*δ*_C_^c^	*δ*_H_ (*J* in Hz) ^b^
2	157.0 C	
3	133.8 C	
4	177.8 C	
5	161.7 C	
6	99.1 CH	6.16 (d, 2.0)
7	164.5 C	
8	94.0 CH	6.36 (d, 2.0)
9	156.9 C	
10	104.5 C	
1′	121.7 C	
2′	116.7 CH	7.52 (d, 2.0)
3′	145.2 C	
4′	148.9 C	
5′	115.7 CH	6.84 (d, 8.0)
6′	122.0 CH	7.53 (dd, 8.0, 2.0)
1″	101.8 CH	5.34 (d, 7.0)
2″	74.5 CH	3.23 (m)
3″	76.9 CH	3.23 (m)
4″	71.8 CH	3.24 (m)
5″	76.3 CH	3.29 (m)
6″	68.0 CH_2_	3.76 (br d, 10.5); 3.33 (overlap)
1‴	101.3 CH	4.41 (br s)
2‴	68.3 CH	3.60 (br s)
3‴	74.7 CH	4.63 (dd, 9.5, 3.5)
4‴	69.4 CH	3.37 (m)
5‴	68.9 CH	3.43 (m)
6‴	18.1 CH_3_	1.03 (d, 6.0)
1⁗	167.3 C	
2⁗	128.1 C	
3⁗	142.0 CH	6.70 (t, 6.5)
4⁗	27.1 CH_2_	2.25 (dt, 6.5, 7.0)
5⁗	38.0 CH_2_	2.07 (t, 7.0)
6⁗	135.6 C	
7⁗	126.1 CH	5.31 (t, 6.5)
8⁗	58.0 CH_2_	3.94 (d, 6.5)
9⁗	16.5 CH_3_	1.61 (s)
10⁗	12.8 CH_3_	1.77 (s)

^a^ Coupling constants (Hz) are given in parentheses. ^b^ Measured at 500 MHz in DMSO-*d*_6_. ^c^ Measured at 100 MHz in DMSO-*d*_6_.

## Data Availability

Data are contained within the article and supplementary materials.

## Supplementary Materials

The following are available online at https://www.mdpi.com/article/10.3390/plants12244083/s1: Table S1: ^1^H and ^13^C NMR Spectrum Data of Compounds **1**–**5**; Figure S1: HR-ESI-MS data of **6**; Figure S2: ^1^H NMR spectrum of **6** in DMSO-*d*_6_ (500 MHz); Figure S3: ^13^C NMR spectrum of **6** in DMSO-*d*_6_ (100 MHz); Figure S4: ^1^H-^1^H COSY spectrum of **6** in DMSO-*d*_6_; Figure S5: HSQC spectrum of **6** in DMSO-*d*_6_; Figure S6: HMBC spectrum of **6** in DMSO-*d*_6_; Figure S7: UV/PDA spectrum of **6**.

## References

[1] Chuang T., Ornduff R. (1992). Seed morphology and systematics of *Menyanthaceae*. AM. J. Bot..

[2] Njuguna A.W., Li Z.-Z., Saina J.K., Munywoki J.M., Gichira A.W., Gituru R.W., Wang Q.-F., Chen J.-M. (2019). Comparative analyses of the complete chloroplast genomes of *nymphoides* and *menyanthes* species (menyanthaceae). Aquat. Bot..

[3] Uesugi R., Tani N., Goka K., Nishihiro J., Tsumura Y., Washitani I. (2005). Isolation and characterization of highly polymorphic microsatellites in the aquatic plant, *Nymphoides peltata* (Menyanthaceae). Mole. Ecol. Notes..

[4] National List of Species of Korea 2022 National Institute of Biological Resources. http://kbr.go.kr/.

[5] Khan Z.R., Chowdhury N.S., Sharmin S., Sohrab M.H. (2018). Medicinal values of aquatic plant genus *Nymphoides* grown in Asia: A review. Asian Pac. J. Trop. Biomed..

[6] Nocchi N., Duarte H.M., Pereira R.C., Konno T.U.P., Soares A.R. (2020). Effects of UV-B radiation on secondary metabolite production, antioxidant activity, photosynthesis and herbivory interactions in *Nymphoides humboldtiana* (Menyanthaceae). J. Photochem. Photobiol. B Biol..

[7] Amin A., Tuenter E., Exarchou V., Upadhyay A., Cos P., Maes L., Apers S., Pieters L. (2016). Phytochemical and pharmacological investigations on *Nymphoides* indica leaf extracts. Phytother. Res..

[8] Murali A., Sudha C., Madhavan V., Yoganarasimhan S. (2007). Anticonvulsant and Sedative Activity of Tagara (*Nymphoides macrospermum*). Pharm. Biol..

[9] Du Y., Wang R., Zhang H., Liu J. (2015). Antitumor constituents of the wetland plant *Nymphoides peltata*: A case study for the potential utilization of constructed wetland plant resources. Nat. Prod. Commun..

[10] Zhou X., Ning K., Ling B., Chen X., Cheng H., Lu B., Gao Z., Xu J. (2019). Multiple injections of autologous adipose-derived stem cells accelerate the burn wound healing process and promote blood vessel regeneration in a rat model. Stem Cells Dev..

[11] Mathias E., Srinivas M.M. (2017). Pediatric thermal burns and treatment: A review of progress and future prospects. Medicines.

[12] Gautam S., Chou C.-F., Dinda A.K., Potdar P.D., Mishra N.C. (2014). Surface modification of nanofibrous polycaprolactone/gelatin composite scaffold by collagen type I grafting for skin tissue engineering. Mater. Sci. Eng. C.

[13] Childs D.R., Murthy A.S. (2017). Overview of wound healing and management. Surg. Clin..

[14] Sun M.-L., Zhao F., Chen X.-L., Zhang X.-Y., Zhang Y.-Z., Song X.-Y., Sun C.-Y., Yang J. (2020). Promotion of wound healing and prevention of frostbite injury in rat skin by exopolysaccharide from the arctic marine bacterium *Polaribacter* sp. SM1127. Mar. Drugs.

[15] Dorai A.A. (2012). Wound care with traditional, complementary and alternative medicine. Indian J. Plast. Surg..

[16] Yeng N.K., Shaari R., Nordin M.L., Sabri J. (2019). Investigation of wound healing effect of *Acalypha indica* extract in sprague dawley rats. Biomed. Pharmacol. J..

[17] Schmidt C.A., Murillo R., Bruhn T., Bringmann G., Goettert M., Heinzmann B., Brecht V., Laufer S.A., Merfort I. (2010). Catechin derivatives from *Parapiptadenia rigida* with in vitro wound-healing properties. J. Nat. Prod..

[18] Tran N.Q., Joung Y.K., Lih E., Park K.D. (2011). In situ forming and rutin-releasing chitosan hydrogels as injectable dressings for dermal wound healing. Biomacromolecules.

[19] Öz B.E., İşcan G.S., Akkol E.K., Süntar İ., Acıkara Ö.B. (2018). Isoflavonoids as wound healing agents from Ononidis Radix. J. Ethnopharmacol..

[20] Muralidhar A., Babu K.S., Sankar T.R., Reddanna P., Latha J. (2013). Wound healing activity of flavonoid fraction isolated from the stem bark of *Butea monosperma* (Lam) in albino wistar rats. Eur. J. Exp. Biol..

[21] Mensah A., Sampson J., Houghton P., Hylands P., Westbrook J., Dunn M., Hughes M., Cherry G. (2001). Effects of *Buddleja globosa* leaf and its constituents relevant to wound healing. J. Ethnopharmacol..

[22] Muhammad A.A., Pauzi N.A.S., Arulselvan P., Abas F., Fakurazi S. (2013). In vitro wound healing potential and identification of bioactive compounds from *Moringa oleifera* Lam. BioMed Res. Int..

[23] Kim T.-Y., Park N.-J., Jegal H., Paik J.-H., Choi S., Kim S.-N., Yang M.H. (2023). *Nymphoides peltata* root extracts improve atopic dermatitis by regulating skin inflammatory and anti-oxidative enzymes in 2, 4-dinitrochlorobenzene (DNCB)-induced SKH-1 hairless mice. Antioxidants.

[24] Blunder M., Orthaber A., Bauer R., Bucar F., Kunert O. (2017). Efficient identification of flavones, flavanones and their glycosides in routine analysis via off-line combination of sensitive NMR and HPLC experiments. Food Chem..

[25] Hardiyanti R., Marpaung L., Adnyana I.K., Simanjuntak P. (2019). Isolation of quercitrin from *Dendrophthoe pentandra* (L.) Miq leaves and it’s antioxidant and antibacterial activities. Rasayan. J. Chem..

[26] Slimestad R., Torskangerpoll K., Nateland H.S., Johannessen T., Giske N.H. (2005). Flavonoids from black chokeberries, *Aronia melanocarpa*. J. Food Compos. Anal..

[27] Iwagawa T., Asai H., Hase T., Sako S., Su R., Hagiwara N., Kim M. (1990). Monoterpenoids from *Radermachia sinica*. Phytochem.

[28] Itoh A., Tanahashi T., Nagakura N., Takenaka Y., Chen C.-C., Pelletier J. (2004). Flavonoid glycosides from *Adina racemosa* and their inhibitory activities on eukaryotic protein synthesis. J. Nat. Prod..

[29] Piipponen M., Li D., Landén N.X. (2020). The immune functions of keratinocytes in skin wound healing. Int. J. Mol. Sci..

[30] Li J., Chen J., Kirsner R. (2007). Pathophysiology of acute wound healing. Clin. Dermatol..

[31] Grada A., Otero-Vinas M., Prieto-Castrillo F., Obagi Z., Falanga V. (2017). Research techniques made simple: Analysis of collective cell migration using the wound healing assay. J. Investig. Dermatol..

[32] Mazereeuw-Hautier J., Gres S., Fanguin M., Cariven C., Fauvel J., Perret B., Chap H., Salles J.-P., Saulnier-Blache J.-S. (2005). Production of lysophosphatidic acid in blister fluid: Involvement of a lysophospholipase D activity. J. Investig. Dermatol..

[33] Mi Y., Zhong L., Lu S., Hu P., Pan Y., Ma X., Yan B., Wei Z., Yang G. (2022). Quercetin promotes cutaneous wound healing in mice through Wnt/*β*-catenin signaling pathway. J. Ethnopharmacol..

[34] Chen J., Li G., Sun C., Peng F., Yu L., Chen Y., Tan Y., Cao X., Tang Y., Xie X. (2022). Chemistry, pharmacokinetics, pharmacological activities, and toxicity of Quercitrin. Phytother. Res..

[35] Süntar I.P., Akkol E.K., Yalçin F.N., Koca U., Keleş H., Yesilada E. (2010). Wound healing potential of *Sambucus ebulus* L. leaves and isolation of an active component, quercetin 3-*O*-glucoside. J. Ethnopharmacol..

[36] Chen L.-Y., Huang C.-N., Liao C.-K., Chang H.-M., Kuan Y.-H., Tseng T.-J., Yen K.-J., Yang K.-L., Lin H.-C. (2020). Effects of Rutin on Wound Healing in Hyperglycemic Rats. Antioxidants.

[37] Velnar T., Bailey T., Smrkolj V. (2009). The wound healing process: An overview of the cellular and molecular mechanisms. J. Int. Med. Res..

[38] Sun Y., Gao M., Chen F., Han R., Du K., Zhang Y., Li M., Si Y., Feng W. (2019). Six New Coumarin Glycosides from the Aerial Parts of *Gendarussa vulgaris*. Molecules.

[39] Lee K.H., Kim J.K., Yu J.S., Jeong S.Y., Choi J.H., Kim J.-C., Ko Y.-J., Kim S.-H., Kim K.H. (2021). Ginkwanghols A and B, osteogenic coumaric acid-aliphatic alcohol hybrids from the leaves of *Ginkgo biloba*. Arch. Pharm. Res..

[40] Yu J.S., Jeong S.Y., Li C., Oh T., Kwon M., Ahn J.S., Ko S.K., Ko Y.J., Cao S., Kim K.H. (2022). New phenalenone derivatives from the Hawaiian volcanic soil-associated fungus *Penicillium herquei* FT729 and their inhibitory effects on indoleamine 2,3-dioxygenase 1 (IDO1). Arch. Pharm. Res..

