# Supplementary material for: 3‴-O-Foliamenthoyl-Rutin, a New Flavonoid Glycoside from the Roots of Nymphoides peltata
Source: Plants (Basel). 2023 Dec 6;12(24):4083. doi: 10.3390/plants12244083 (PMC10748241; doi:10.3390/plants12244083)

Supplementary Materials

3‴-*O*-Foliamenthoyl-Rutin, a New Flavonoid Glycoside from the Roots of *Nymphoides peltata*

Tae-Young Kim [1,†], Bum Soo Lee [2,†], Beom-Geun Jo [1,†], Seong Pil Heo [3], Min-Ji Keem [1], Taek-Hwan Kwon [1], Su-Nam Kim [3,4,*], Ki Hyun Kim [2,*] and Min Hye Yang [1,*]

[1] Department of Pharmacy, College of Pharmacy and Research Institute for Drug Development, Pusan National University, Busan 46241, Republic of Korea; taeyour@pusan.ac.kr (T.-Y.K.); bg_jo@pusan.ac.kr (B.-G.J.); mj_keem@pusan.ac.kr (M.-J.K.); thkwon@pusan.ac.kr (T.-H.K.)

[2] School of Pharmacy, Sungkyunkwan University, Suwon 16419, Republic of Korea; kosboybs@skku.edu

[3] Natural Products Research Institute, Korea Institute of Science and Technology, Gangneung 25451, Republic of Korea; 123045@kist.re.kr

[4] Division of Bio-Medical Science and Technology, KIST School, University of Science and Technology, Seoul 02792, Republic of Korea

* Correspondence: snkim@kist.re.kr (S.-N.K.); khkim83@skku.edu (K.H.K.); mhyang@pusan.ac.kr (M.H.Y.); Tel.: +82-31-290-7700 (K.H.K.)

† These authors contributed equally to this study.

Contents of Supplementary material

Table S1. ^{1}H and ^{13}C NMR Spectrum Data of Compounds **1-5**

Figure S1. HR-ESI-MS data of **6**

Figure S2. ^{1}H NMR spectrum of **6** in DMSO-d_6 (500 MHz)

Figure S3. ^{13}C NMR spectrum of **6** in DMSO-d_6 (100 MHz)

Figure S4. ^{1}H-^{1}H COSY spectrum of **6** in DMSO-d_6

Figure S5. HSQC spectrum of **6** in DMSO-d_6

Figure S6. HMBC spectrum of **6** in DMSO-d_6

Figure S7. UV/PDA spectrum of **6**

Table S1. ^{1}H and ^{13}C NMR Spectrum Data of Compounds **1-5**

Position	Compound **1**		Compound **2**		Compound **3**		Compound **4**		Compound **5**	
	δ_C, type [c]	δ_H (J in Hz) [b]	δ_C, type [c]	δ_H (J in Hz) [a]	δ_C, type [c]	δ_H (J in Hz) [a]	δ_C, type [c]	δ_H (J in Hz) [a]	δ_C, type [c]	δ_H (J in Hz) [a]
2	145.9 C		156.6 C		156.8 C		156.8 C		159.1 C	
3	134.8 C		134.1 C		133.8 C		133.8 C		135.7 C	
4	174.9 C		177.2 C		177.9 C		177.8 C		179.8 C	
5	159.8 C		157.3 C		161.7 C		161.7 C		163.6 C	
6	102.1 CH	6.16 (d, 2.0)	100.6 CH	6.15 (d, 2.0)	99.2 CH	6.20 (d, 2.0)	99.2 CH	6.14 (d, 2.0)	101.1 CH	6.15 (d, 2.0)
7	163.0 C		161.6 C		164.8 C		164.7 C		166.7 C	
8	92.4 CH	6.38 (d, 2.0)	94.8 CH	6.34 (d, 2.0)	94.0 CH	6.40 (d, 2.0)	94.0 CH	6.34 (d, 2.0)	96.0 CH	6.34 (d, 2.0)
9	155.2 C		156.6 C		156.6 C		156.8 C		158.8 C	
10	97.3 C		102.4 C		104.4 C		104.5 C		106.3 C	
1′	121.0 C		121.5 C		122.1 C		122.1 C		123.5 C	
2′	114.7 CH	7.65 (d, 2.0)	115.9 CH	7.49 s	116.7 CH	7.61-7.53 (m)	116.6 CH	7.53 (s)	118.7 CH	7.49 (s)
3′	144.1 C		146.1 C		145.3 C		145.3 C		147.2 C	
4′	146.8 C		146.1 C		149.0 C		149.0 C		150.9 C	
5′	114.7 CH	6.86 (d, 8.5)	115.9 CH	6.80 (d, 8.0)	115.7 CH	6.84 (d, 9.0)	115.8 CH	6.81 (d, 9.0)	117.7 CH	6.80 (d, 8.0)
6′	119.1 CH	7.52 (dd, 8.5, 2.0)	121.5 CH	7.50 (d, 8.0)	121.6 CH	7.61-7.53 (m)	121.6 CH	7.54 (d, 2.0)	124.0 Ch	7.51 (d, 2.0)
1″			102.2 CH	5.30 (d, 7.5)	101.4 CH	5.46 (d, 7.5)	101.3 CH	5.33 (d, 7.5)	103.6 CH	5.30 (d, 7.5)
2″			70.8 CH	4.90 (m)	74.6 CH	3.03 (m)	72.9 CH	2.92 (m)	76.5 CH	3.18 (m)
3″			71.0 CH	3.99 (m)	78.11 CH	3.21 (m)	77.4 CH	3.25 (m)	78.9 CH	3.17 (m)
4″			71.7 CH	3.52 (m)	70.4 CH	3.17 (m)	70.5 CH	3.04 (m)	72.4 CH	3.30 (m)
5″			70.6 CH	3.22 (m)	77.0 CH	3.04 (m)	76.8 CH	3.18 (m)	78.3 CH	3.20 (m)
6″			18.0 CH_3	0.95 (d, 6.0)	61.5 CH_2	3.58 (d, 11.5), 3.28 (overlap)	67.7 CH_2	3.73 (d, 11.0), 3.41 (overlap)	69.4 CH_2	3.66 (d, 10.0), 3.24 (overlap)
1‴							103.2 CH	3.91 (d, 6.5)	103.2 CH	4.34 (d, 1.5)
2‴							70.9 CH	3.10 (m)	70.7 CH	3.23 (m)
3‴							74.4 CH	3.19 (m)	74.3 CH	3.03 (m)
4‴							67.8 CH	3.36 (m)	72.9 CH	3.25 (m)
5‴							65.3 CH_2	3.42 (overlap), 2.85 (dd, 12.0, 2.0)	72.8 CH	3.36 (m)
6‴									20.2 CH_3	0.95 (d, 6.0)

[a] Measured at 400 MHz in DMSO-d_6. [b] Measured at 500 MHz in DMSO-d_6. [c] Measured at 100 MHz in DMSO-d_6.

Figure S1. HR-ESI-MS data of **6**

Compound Identification Results: Cpd 663: C37 H44 O18; 4.612

ID Techniques Applied
MFG

Best	Name	Formula	m/z	Mass	Species	Diff (ppm)	RT	Score
⦿		C37 H44 O18	777.2561 799.2405	776.251	(M+H)+ (M+Na)+	-2.29	4.612	94.7
○		C44 H40 O13	777.2561 799.2405	776.251	(M+H)+ (M+Na)+	5.23	4.612	76.14
○		C26 H48 O26	777.2561	776.2499	(M+H)+	8.41	4.612	56.61

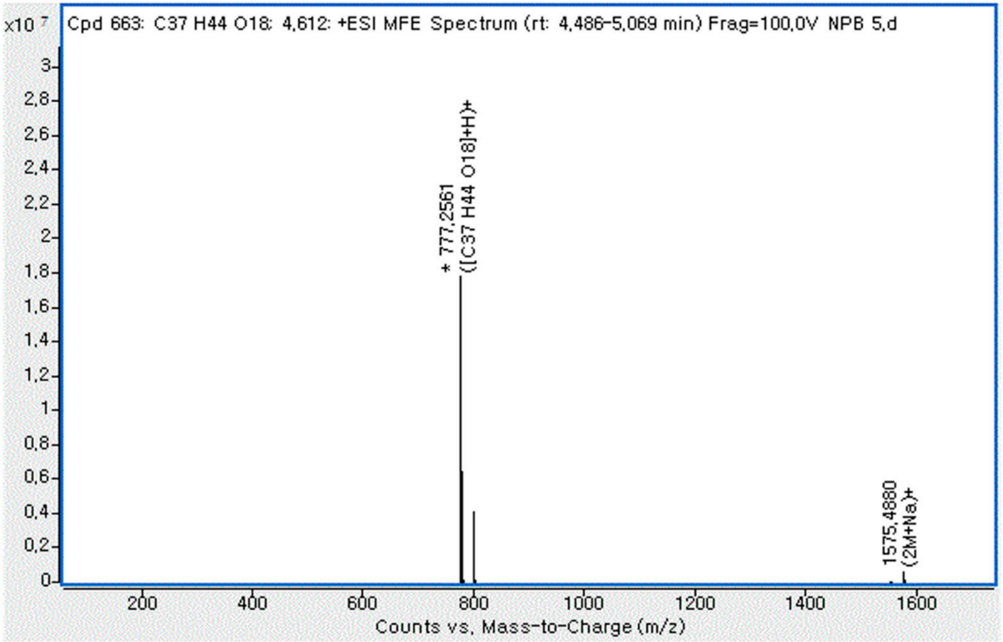

Figure S2. ^{1}H NMR spectrum of **6** in DMSO-d_6 (500 MHz)

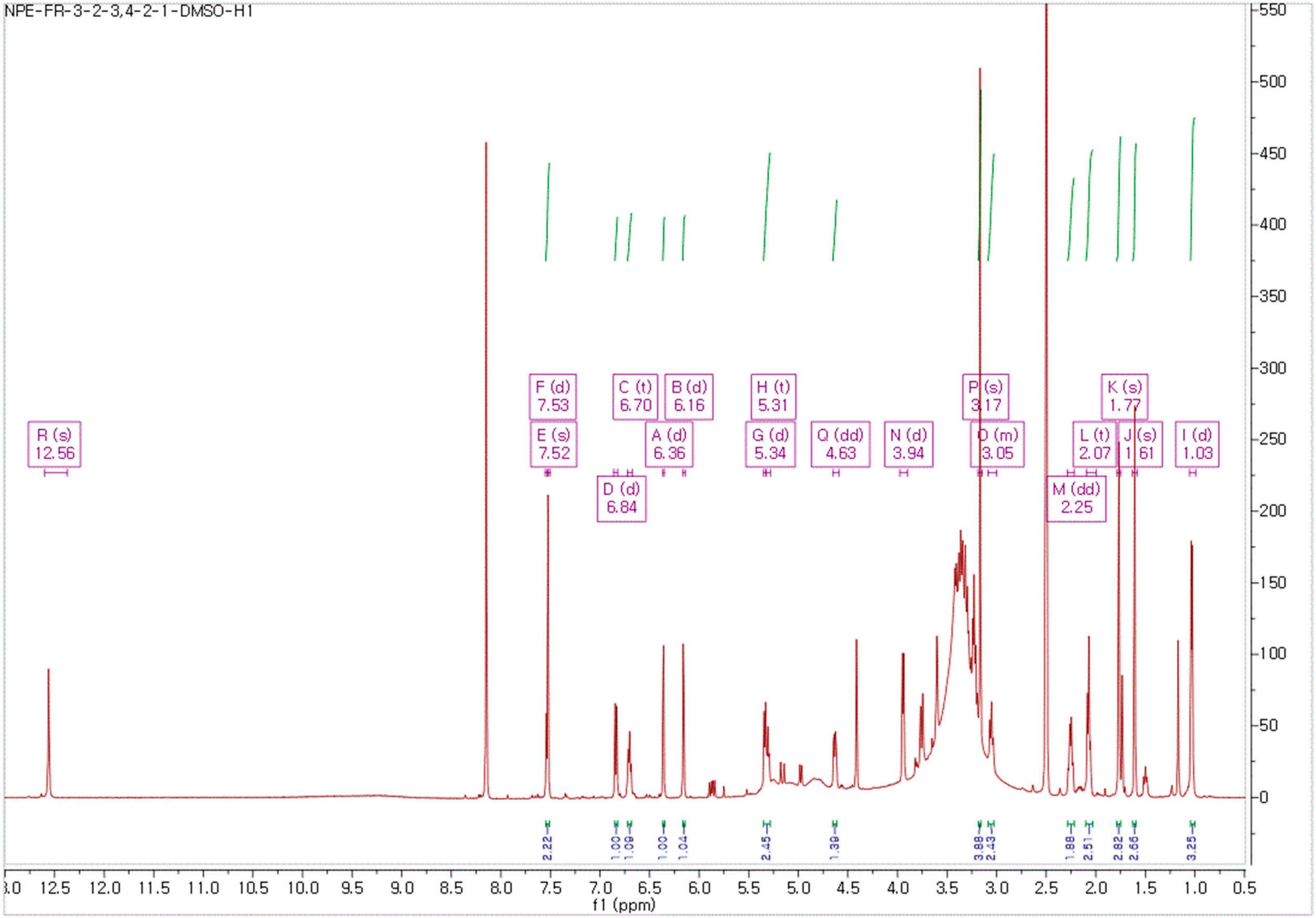

Figure S3. ^{13}C NMR spectrum of **6** in DMSO-d_6 (100 MHz)

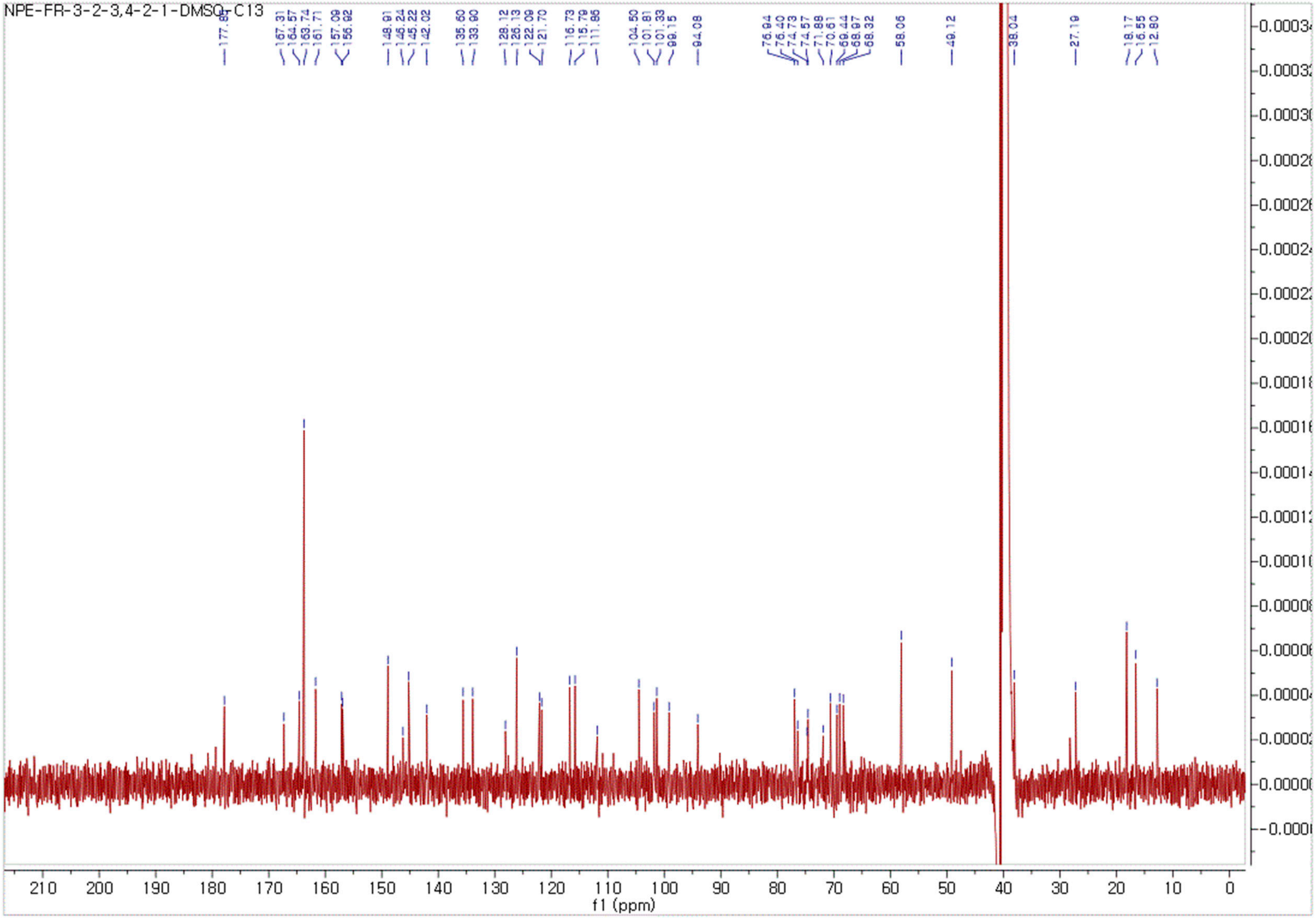

Figure S4. ^{1}H-^{1}H COSY spectrum of **6** in DMSO-d_6

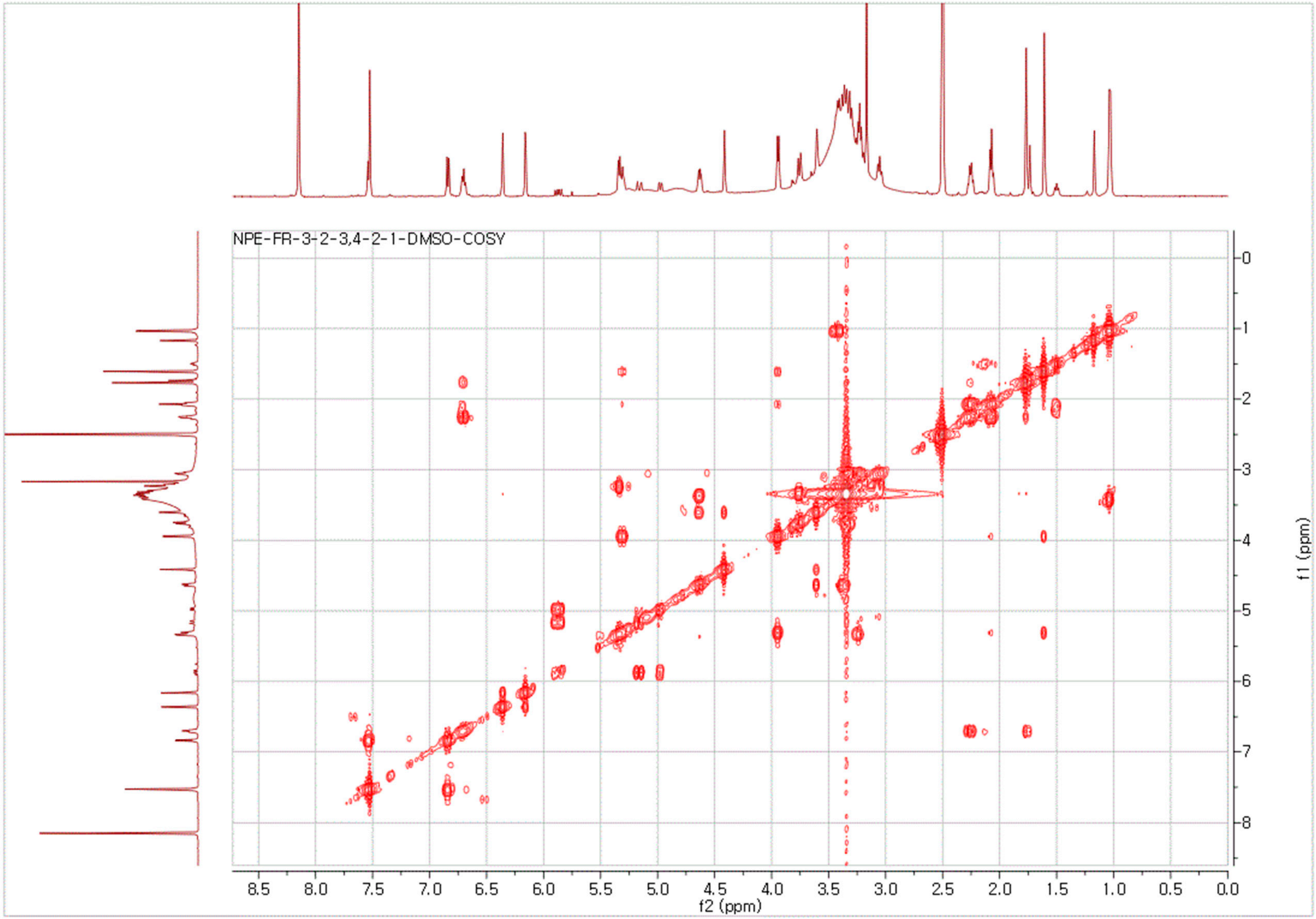

Figure S5. HSQC spectrum of **6** in DMSO-d_6

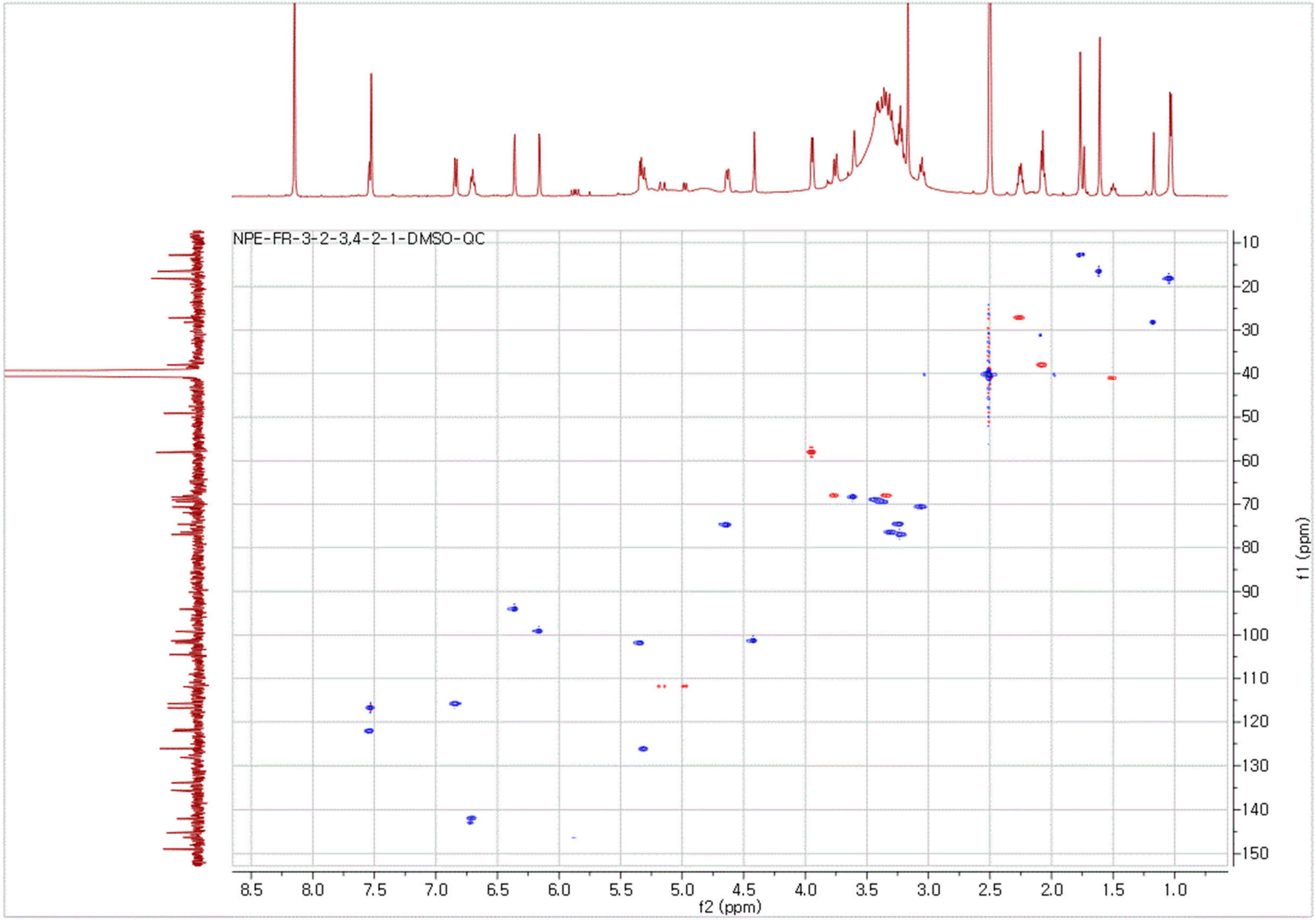

Figure S6. HMBC spectrum of **6** in DMSO-d_6

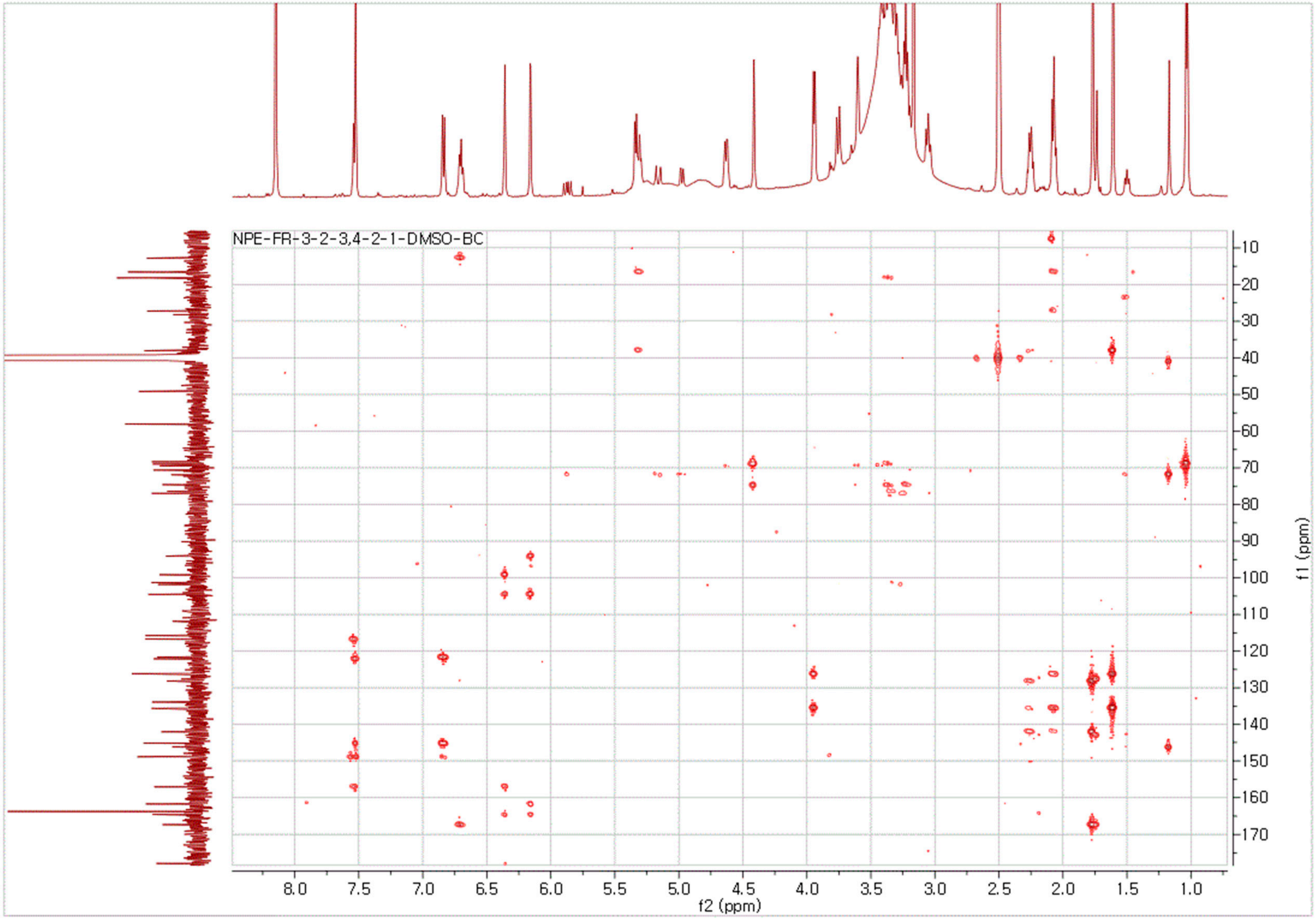

Figure S7. UV/PDA spectrum of **6**

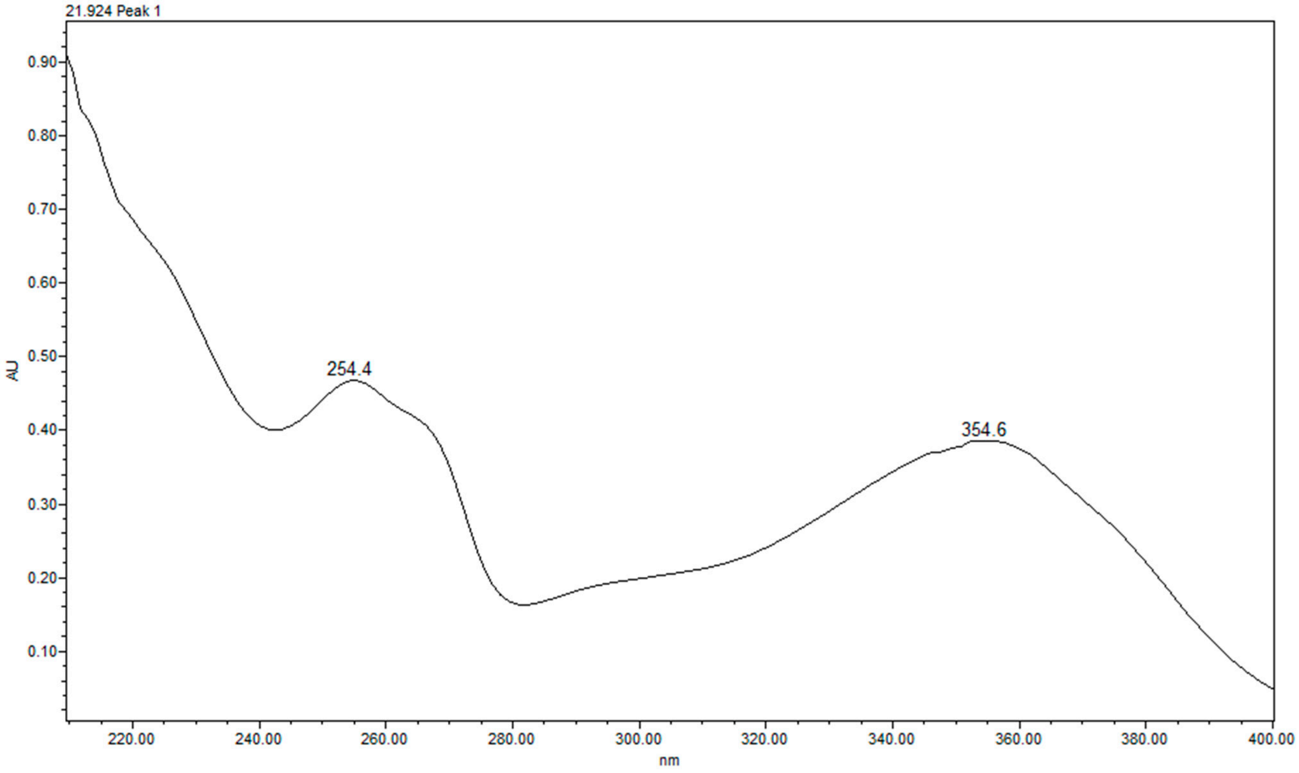

Supplement: Supplementary file 1 [file plants-12-04083-s001.zip › plants-2723536-supplementary.pdf]
